# Visualizing Single Molecular Crystals by Electrochemiluminescence Microscopy

**DOI:** 10.1021/cbmi.5c00184

**Published:** 2025-10-24

**Authors:** Yufei Wang, Jianping Lei

**Affiliations:** State Key Laboratory of Analytical Chemistry for Life Science, School of Chemistry and Chemical Engineering, 12581 Nanjing University, Nanjing 210023, China

Electrochemiluminescence (ECL) is a light-emitting process driven by electrochemical reactions near the electrode surface, first pioneered by Bard’s group in 2002.^1^ Due to the absence of external light excitation, the ECL technique enables an excitation–emission decoupling capability with remarkable advantages such as near-zero background, high sensitivity, and controllable spatial resolution. By integrating high-resolution microscopy with ECL signal transduction, ECL microscopy originating directly from electrochemical reactions serves as a spatiotemporal imaging tool for studying ECL reaction kinetics and visualizing single particles, even down to single molecules.^2^ However, the spatially random reaction between conventional ECL luminophores and coreactants in solution often leads to low photon collection efficiency and diminished ECL output.

Recent advances have shown that single crystals, with high crystallinity and structural order with atomic-scale precision, can function as efficient ECL emitters,^3,4^ providing the periodic lattice necessary for efficient long-range energy transfer and directed photon propagation. The successful implementation of single crystals in ECL microscopy requires meeting a stringent set of material criteria: (1) The crystals must have robust electrochemical stability to withstand the applied potentials without degradation, ensuring sustainable and reproducible ECL emission. (2) High ECL efficiency triggered by electrochemistry is desired and depends on the intrinsic photophysics of the luminophore, governed by a competition between charge- and energy-transfer pathways within the crystalline lattice. (3) Morphological control is critical. Crystals should be engineered with a defined size, aspect ratio, and surface flatness to ensure optimal contact with the electrode, efficient charge injection, and compatibility with optical microscopy setups. It is the intricate interplay of these factors—structural integrity, electrochemical robustness, emission efficiency, and tailored morphology—that enables a mere crystalline material to function as a high-performance nanoemitter for advanced ECL imaging.^5^

Reticular materials, such as metal–organic frameworks (MOFs) and covalent organic frameworks, are molecular crystalline materials constructed from the ordered arrangement of organic ligands and/or metal atoms.^6–8^ Due to the predesignable structure and long-range charge transfer capacity, reticular materials offer a uniquely programmable platform for constructing crystalline ECL emitters. In a recent report in *Angewandte Chemie*, Lei and colleagues introduced an emerging polychromatic ECL imaging system using single MOF crystals, in which the morphological distribution of porphyrin can be dynamically controlled at the specific sites of single crystals. A high-performance ECL system of MOF crystal was engineered based on a magnesium­(II) node, suppressing ligand-to-metal charge transfer quenching, and pyrene-based ligands, which are renowned for efficient ECL (Figure 1a).^9^ The parent structure, NJU-241, features a Mg–carboxylate building unit that enforces a critical interligand distance of 0.624 nm between pyrene cores (Figure 1b). This precise spacing suppresses aggregation-caused quenching while permitting efficient energy transfer. Building on this optimized monochromatic emitter, researchers constructed the heteroligand NJU-241 (h-NJU-241) by strategically incorporating porphyrin acceptors (Figure 1a). The energy transfer efficiency from pyrene to porphyrin was found to be critically dependent on the porphyrin incorporation ratio, reaching a remarkable 92.2% at a doping level of 1.87‰. In the presence of tripropylamine as a coreactant, the MOF crystal displayed exceptionally bright and stable ECL emission. Moreover, the ECL spectra of h-NJU-241 exhibited a gradual decrease in intrinsic blue emission from pyrene and a concurrent increase in red emission from porphyrin as the coordination ratio of porphyrin increased (Figure 1c), suggesting a high intrareticular energy transfer efficiency between pyrene and porphyrin in the MOF crystal.

**Figure 1:**
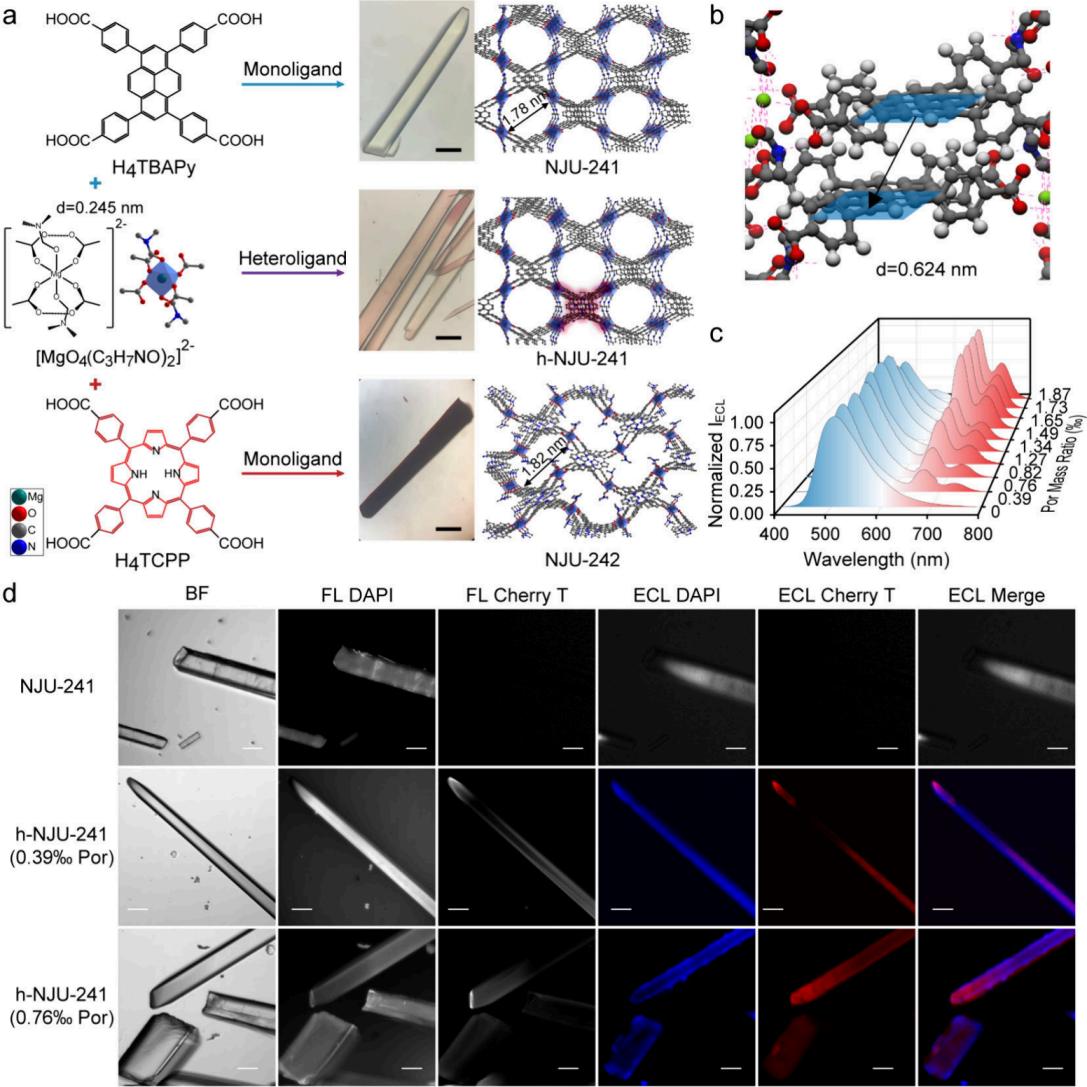
(a) Schematic diagrams of the preparation of Mg-MOF emitters with single crystal images and top view structures. (b) Schematic illustration of the pyrene–pyrene distance in the NJU-241 unit. (c) ECL spectra of h-NJU-241 with a porphyrin ratio of 0 to 1.87‰. (d) Images of bright-field, fluorescence DAPI and Cherry-T channels, and ECL DAPI and Cherry-T channels captured by EMCCD for NJU-241, h-NJU-241 (0.39‰ por), and h-NJU-241 (0.76‰ por). Scale bars: 100 μm. Reproduced with permission from ref 9. Copyright 2025 Wiley-VCH.

Leveraging the precise spectral tuning and stable ECL characteristics of heteroligand MOFs, these single crystals can be utilized for polychromatic ECL imaging. Both NJU-241 and h-NJU-241 form regularly shaped rodlike crystals with high crystallinity, appropriate thickness, low electrochemical impedance, and high transparency, making them suitable for ECL imaging. By applying a super-resolution radial fluctuation algorithm for image correction, bright ECL images of h-NJU-241 single crystals were obtained with the enhanced grayscale contrast and resolution. This efficient cascade harnesses the brilliant ECL of the pyrene donor and converts it into strong, stable red emission from the porphyrin acceptor, enabling a single h-NJU-241 crystal to function as a dual-color ECL source under one step potential and pioneering polychromatic ECL imaging at the single-crystal level (Figure 1d). Furthermore, by adjusting the reaction conditions, the specific sites of porphyrin within the MOF skeleton can be dynamically controlled, providing a tailored crystalline platform for ECL microscopy.

Beyond serving as multiplexed light sources, single crystals can be engineered to fundamentally redefine the spatial paradigm of ECL. A foundational insight came from the work of Su and colleagues, who demonstrated that hexagonal single crystals of iridium­(III) complexes function as intrinsic active optical waveguides. This discovery revealed that such crystals could efficiently transmit self-generated ECL signals over remarkable distances exceeding 100 μm, achieving a 5-fold amplification of emission intensity at remote terminals and establishing the core principle of excitation–emission decoupling.^10^ Furthermore, this principle was rigorously quantified using a patterned three-band electrode array for spatially selective excitation, which enabled precise mapping of ECL propagation (Figure 2a). Through crystal engineering, both solid molecular crystals (sMCs) and microrod-shaped crystals featuring terminal microcavities (mMCs) were optimized. Compared to sMCs, which exhibited significant optical loss (up to 93%), the mMCs drastically reduced waveguide loss to as low as 15%. Most impressively, under a middle-in/terminal-out excitation mode, the microcavities enabled an up to 6.8-fold enhancement of terminal output intensity—a transformative effect not observed in sMCs (Figure 2b,c).^11^ This evolution from a unidirectional waveguide to an active, amplifying optical component pushes the spatial decoupling concept into a practical tool for remote imaging with enhanced signal collection.

**Figure 2:**
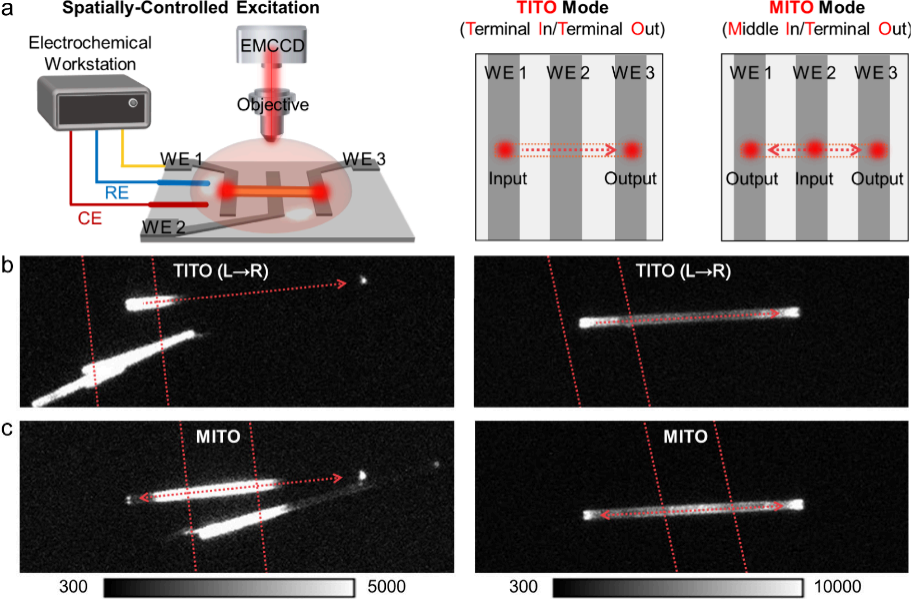
(a) Schematic illustration of the setup for studying ECL waveguiding performance and spatially selective electrochemical excitation by terminal-in/terminal-out and middle-in/terminal-out modes. (b, c) ECL images of single sMCs (left panel) and mMCs (right panel) deposited on the band-shaped ITO electrode array. Scale bars: 20 μm. L → R indicates the propagation direction of ECL from left to right. Reproduced from ref 11. Copyright 2025 American Chemical Society.

The convergence of advanced crystalline emitters with computation and instrumentation is poised to unlock a new frontier in ECL microscopy. The stochastic, multidimensional data from ECL are ideal for machine learning.^12,13^ Machine learning algorithms can reconstruct super-resolved images and, crucially, decode complex spatiotemporal dynamics. This is particularly powerful when combined with ultrahigh-speed CCD imaging, which can capture ECL signals with millisecond temporal resolution. This combination enables the *in situ* visualization of electrochemical reaction kinetics, such as mapping transient active sites on a single catalyst particle or tracking biological transport in real time.^14–16^ Moreover, realizing this vision will require innovative instrumentation, including integrated and miniaturized ECL microscopes. Such systems could combine microfabricated electrode arrays with optical paths dedicated to collecting waveguided light, making high-resolution ECL analysis accessible for complex environments like live-cell studies or lab-on-a-chip devices.

In conclusion, beyond traditional molecular luminophores with isotropic emission and spatial blurring, a paramount advantage of single molecular crystals is their capacity for efficient long-range energy transfer for high-resolution imaging. The long-range, waveguided ECL emission of crystalline nanoemitters enables a revolutionary concept: remote ECL sensing. That is, the crystalline nanoemitter can be positioned at a target site, and upon remote electrochemical triggering, the optical signal would be propagated back through the crystal waveguide, efficiently delivering ECL signals deep into biological samples for *in situ* analysis. Overall, single-crystal-based ECL microscopy holds great potential for visualizing and understanding fundamental dynamics in chemical and biological systems.
